# Real-time assessment of right and left ventricular volumes and function in children using high spatiotemporal resolution spiral bSSFP with compressed sensing

**DOI:** 10.1186/s12968-018-0500-9

**Published:** 2018-12-06

**Authors:** Jennifer A. Steeden, Grzegorz T. Kowalik, Oliver Tann, Marina Hughes, Kristian H. Mortensen, Vivek Muthurangu

**Affiliations:** 1 UCL Centre for Cardiovascular Imaging, Institute of Cardiovascular Science, University College London, 30 Guildford Street, London, WC1N 1EH UK; 2 Cardiorespiratory Unit, Great Ormond Street Hospital for Children, London, WC1N 3JH UK

**Keywords:** Real-time, Left ventricular volumes, Right ventricular volumes, Spiral, Compressed sensing, Paediatric, Children, Congenital heart disease

## Abstract

**Background:**

Real-time cardiovascular magnetic resonance (CMR) assessment of ventricular volumes and function enables data acquisition during free-breathing. The requirement for high spatiotemporal resolution in children necessitates the use of highly accelerated imaging techniques.

**Methods:**

A novel real-time balanced steady state free precession (bSSFP) spiral sequence reconstructed using Compressed Sensing (CS) was prospectively validated against the breath-hold clinical standard for assessment of ventricular volumes in 60 children with congenital heart disease. Qualitative image scoring, quantitative image quality, as well as evaluation of biventricular volumes was performed. Standard BH and real-time measures were compared using the paired t-test and agreement for volumetric measures were evaluated using Bland Altman analysis.

**Results:**

Acquisition time for the entire short axis stack (~ 13 slices) using the spiral real-time technique was ~ 20 s, compared to ~ 348 s for the standard breath hold technique. Qualitative scores reflected more residual aliasing artefact (*p* < 0.001) and lower edge definition (*p* < 0.001) in spiral real-time images than standard breath hold images, with lower quantitative edge sharpness and estimates of image contrast (*p* < 0.001).

There was a small but statistically significant (*p* < 0.05) overestimation of left ventricular (LV) end-systolic volume (1.0 ± 3.5 mL), and underestimation of LV end-diastolic volume (− 1.7 ± 4.6 mL), LV stroke volume (− 2.6 ± 4.8 mL) and LV ejection fraction (− 1.5 ± 3.0%) using the real-time technique. We also observed a small underestimation of right ventricular stroke volume (− 1.8 ± 4.9 mL) and ejection fraction (− 1.4 ± 3.7%) using the real-time imaging technique. No difference in inter-observer or intra-observer variability were observed between the BH and real-time sequences.

**Conclusions:**

Real-time bSSFP imaging using spiral trajectories combined with a compressed sensing reconstruction showed good agreement for quantification of biventricular metrics in children with heart disease, despite slightly lower image quality. This technique holds the potential for free breathing data acquisition, with significantly shorter scan times in children.

**Electronic supplementary material:**

The online version of this article (10.1186/s12968-018-0500-9) contains supplementary material, which is available to authorized users.

## Background

Evaluation of biventricular volumes and function is vital in the investigation of children with congenital heart disease. The clinical standard method of evaluating ventricular volumes is cardiovascular magnetic resonance (CMR) – specifically, multi-slice cardiac gated balanced steady state free precession (bSSFP) cine imaging [1]. This technique is now routinely used in children and has an important role to play in disease management [2]. Unfortunately, it does require multiple breath-holds (BH) and this can be difficult in young children with heart disease. An alternative approach is real-time CMR in which each *k*-space is acquired in a single shot manner, rather than over several heart beats. The benefit of real-time CMR is that it can be acquired during free breathing and without cardiac gating. However, this comes at the cost of lower spatial and temporal resolution, which can affect accuracy [3].

One solution is to leverage accelerated imaging techniques (data undersampling or more efficient *k*-space filling) to improve resolution. Recently, a combination of undersampled radial *k*-space filling and compressed sensing (CS) has been shown to enable relatively high resolution real-time imaging [4, 5]. Studies in adults have demonstrated good agreement between these radial real-time techniques and the breath-hold clinical standard for assessment of ventricular volumes [6–9]. However, imaging children requires much higher spatial and temporal resolution than available using conventional radial CS methods. Hence greater acceleration is needed to translate these techniques into the paediatric population. One possibility is to combine CS with more efficient spiral *k*-space filling. Spiral trajectories have previously been used to accelerate real-time phase contrast CMR with some success [10]. They can also be combined with golden angle spacing [11, 12] to enforce incoherent aliasing necessary for CS reconstruction.

In this study, we implemented a novel real-time bSSFP spiral sequence reconstructed using CS. The purpose of this study was to validate this real-time sequence against the BH clinical standard for assessment of biventricular volumes in children with heart disease.

## Methods

### Study population

Between December 2017 and February 2018, 60 consecutive children referred for CMR and consented for additional research scans were included into this study. The local committee of the UK National Research Ethics Service approved the study (06/Q0508/124), and written consent was obtained from all subjects/guardians. Patients were excluded from this study if: a) there was a requirement for general anesthetic (15 children excluded during the recruitment period), b) the subject had an irregular heart rate or difficulty performing breath-holds (11 children excluded), or c) the subject had single ventricular anatomy which was excluded to ensure that biventricular volumes could be evaluated in all patients (4 children excluded).

### Imaging protocol

All imaging was performed on a 1.5 Tesla CMR scanner (Avanto, Siemens Healthineers, Erlangen, Germany) with maximum gradient amplitude of 40 mT/m and a maximum slew rate of 180 T/m/s. Signal detection was accomplished using two spine coils incorporated into the scan table and arranged in head-foot manner, and one anteriorly placed body-matrix coil (giving a total of 12 coil elements). A vector electrocardiographic system was used for cardiac gating.

#### Standard volumetric assessment

Standard cardiac-gated ventricular volume assessment was performed using a multi-slice retrospectively cardiac gated bSSFP Cartesian sequence. The temporal resolution was 29.5 ms and the in-plane spatial resolution was 1.5 × 1.5 mm (full sequence parameters are shown in Table 1). The imaging plane was in the ventricular short axis (SAX), planned using right ventricular (RV) and left ventricular (LV) long axes and 4-chamber images. Sufficient contiguous slices were acquired in the short axis to ensure coverage of the whole ventricle (~ 13 ± 2 slices, range: 10 to 17). Each slice was acquired in a separate breath-hold, each lasting ~ 5.5 ± 1.1 s (range: 3.6 to 8.8 s).

**Table 1:** Sequence parameters

	Standard BH technique	Spiral real-time technique
Field of view (mm)	350	350
Rectangular field-of-view (%)	75	100
Matrix size	240 × 180	208 × 208
Number of slices	~ 13 (range: 10–17)	~ 13 (range: 10–17)
Image resolution (mm)	1.46 × 1.46 × 8	1.68 × 1.68 × 8
TE/TR (ms)	1.16/2.32	0.67/3.34
Flip Angle (degree)	67	67
Pixel Bandwidth (Hz/pixel)	1225	1502
Cardiac gating	Retrospective	–
Temporal resolution (ms)	29.48	30.06
Reconstructed cardiac phases	40	~ 24 (range: 20–37)
Trajectory	Cartesian	Spiral
Fully sampled spiral interleaves	–	72
GRAPPA	×2	–
Compressed Sensing	–	×8
Breath-hold time per slice (sec)	3.6–8.8	–
Total acquisition time for SAX (sec)	~ 350 (range: 239–573)	~ 20 (range: 11 to 36)

BH, breath hold; TE, echo time; TR, repetition time, GRAPPA, generalized autocalibrating partially parallel acquisition

#### Real-time volumetric assessment

**Fig. 1:**
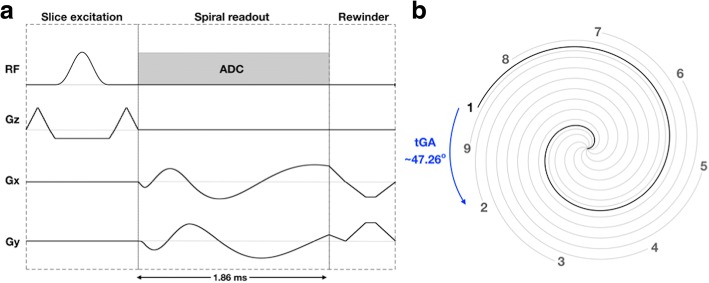
Pulse sequence diagram and k-space trajectory design. **a** Pulse sequence diagram for the spiral real-time balanced steady state free precession (bSSFP) technique. *TE =* 0.67 ms, TR = 3.34 ms. The refocusing nulls the zeroth moments of the gradient waveform only, **b** *k*-space trajectory, showing 9 consecutive TRs. The angle increment between successive spoke numbers is constant with an angle of ${360}^o/\left(6+\frac{\left(1+\sqrt{5}\right)}{2}\right)$ ≈ 47.26^o^

Real-time bSSFP imaging was performed using a 2D multi-slice uniform density spiral sequence. The spiral trajectories were designed using the method described by Hargreaves [13], assuming a field-of-view of 350 mm, a spatial resolution of ~ 1.7 × 1.7 mm and 72 regularly spaced spiral interleaves for complete filling of *k*-space. A zeroth moment rewinder gradient was added at the end of the spiral readout to maintain bSSFP coherence (see Fig. 1 for pulse sequence diagram). In this study we did not use bipolar first moment rewinder gradients, due to the associated increase in TR [14]. Consecutive interleaves were rotated by the tiny golden angle (tGA, ~ 47.26^o^) throughout data acquisition. This enforced incoherent aliasing required for the CS reconstruction, whilst being associated with lower eddy currents than traditional golden angle sampling patterns [15]. Each real-time frame was formed by combining 9 consecutive spiral interleaves (acceleration factor = 8), resulting in a temporal resolution of ~ 30 ms. The full sequence parameters are shown in Table 1.

Each slice was acquired over two R-R intervals. The first was used to reach the steady state and the second was used for acquisition of data. Synchronisation of acquisition of data to the start of the R-wave for each slice also ensured the real-time images were temporally aligned for measurement purposes. After acquisition of each slice, the imaging plane was moved to the next contiguous slice. The imaging plane and the number of slices was the same as the clinical standard BH sequence. All real-time image acquisition was performed during free breathing.

The CS reconstruction was performed on an external GPU equipped computer (Tesla K40c, NVIDIA Corportation, Santa Clara, California, USA) with on-line communication to the native reconstructor [16, 17]. Our implementation of CS solved a set of non-linear equations (representing the imaging process) through minimization of the following cost function:

$$ \underset{\rho }{argmin}\left\{\parallel E\rho -y{\parallel}_2^2+{\lambda}_1\parallel T{V}_t\rho {\parallel}_1\right\} $$

The first term enforces data consistency - where *ρ* is image data, *E* is the encoding matrix (the multi-coil non-uniform Fourier transform (FT) operator) and *y* is the acquired *k*-space data. The second term enforces sparse results through L_1_ norm regularization. A temporal finite difference operator (*TV*_*t*_), was used transform the image data into a sparser representation. This is similar to the previously described iterative Golden-angle Radial Sparse Parallel (GRASP) reconstruction [18]. The optimization was performed using a non-linear Conjugate Gradient algorithm, with 35 iterations. The regularization parameter (λ) were selected empirically on the first 10 patients, to optimize artefact removal without temporal blurring and was set at 0.05 for all patients. As the data for each slice is independent, reconstruction started immediately after the last phase for each slice. Coil sensitivity information was calculated from the time-averaged data (from each slice) [19].

### Image processing and scoring

Evaluation of ventricular volumes and qualitative image scoring was performed by three clinical CMR specialists (OT – 12 years experience, MH – 11 years experience, and KM – 5 years experience). For both image quality scoring and assessment of LV and RV volumes, each clinician was the primary observer for 20 unique cases, of which 10 were re-evaluated to assess intra-observer variability. In addition, each observer assessed 10 cases from a different primary observer (5 cases from each of the other observers), to evaluate inter-observer variability. Thus, each observer scored and processed 40 cases. Overall 30 cases were used to evaluate intra-observer variability and the other 30 cases used to evaluate inter-observer variability.

#### Image quality

The mid-ventricular short-axis cine loops from each technique were scored on a 5-point Likert scale in three categories: sharpness of endocardial border (1 = non-diagnostic, 2 = poor, 3 = adequate, 4 = good, 5 = excellent), temporal fidelity (or blurring) of wall motion (1 = non-diagnostic, 2 = poor, 3 = adequate, 4 = good, 5 = excellent) and residual aliasing artefacts, which appear as spiral streaks across image (1 = non-diagnostic, 2 = severe, 3 = moderate, 4 = mild, 5 = minimal). Each observer scored the patient data that they had previously segmented (*N* = 40 cases each), at a separate session. All loops were presented in a random manner using a custom-built python application, with the observers blinded to diagnosis, patient number and type of sequence.

#### Ventricular function

Quantification of LV and RV volumes was performed in a similar manner for each technique using the OsiriX open source DICOM viewing platform (OsiriX v.9.0, OsiriX Foundation, Geneva, Switzerland) [20]. Firstly, the end-diastolic and end-systolic phases were identified for each ventricle through visual inspection of the mid-ventricular cine. The endocardial borders of all slices at end systole and diastole were then traced manually (including papillary muscles and trabeculation in the myocardial mass). This allowed calculation of end-diastolic volume (EDV) and end-systolic volume (ESV). Stroke volume (SV) was obtained by subtracting ESV from EDV and ejection fraction (EF) = SV/EDV × 100. In addition, LV epicardial borders were traced in end-diastole and combined with endocardial borders to obtain LV mass. Observers were presented with each anonymized volume (including repeated volumes for intra-observer variability) in a random order, blinded to diagnosis, patient number and type of sequence.

### Quantitative image assessment

Calculation of signal-to noise ratio (SNR) and contrast-to-noise ratio (CNR) in images reconstructed using CS is nontrivial due to the uneven distribution of noise. Therefore, we calculated blood pool-to-myocardial signal intensity ratio [3] as a quantitative measure of image contrast, which is important when segmenting data. In all patients, the average blood pool and myocardial signal intensity values were measured in the end diastolic volume (from the LV endocardial and LV epicardial borders drawn for volumetric calculation, across the entire volume). The image contrast equalled blood pool signal intensity divided by myocardial signal intensity.

Edge sharpness of the mid-ventricular short-axis slice was evaluated by measuring the maximum gradient of the normalized pixel intensities across the border of the septum, as previously described [21]. To reduce noise, which results in artificially high gradients (representing sharp edges), the pixel intensities were fit to a tenth order polynomial, before differentiation. Edge sharpness was calculated in six positions across the septum, for all cardiac phases, and the average value was used for comparison.

### Statistics

Statistical analyses were performed by using STATA software (STATA SE, v.14.2, Stata Corporation, College Station, Texas, USA). All results are expressed as the mean ± standard deviation. Mean ventricular volumes, function and mass measured using the standard BH and spiral real-time techniques were compared using a paired t-test. For assessment of agreement of ventricular volumes and function, the standard BH data was used as the clinical standard for Bland-Altman analysis. Association of the percentage differences between the real-time and BH data against both heart rate and body surface area were tested using Spearman’s rank correlation coefficient. Inter and intra-observer variability was assessed using the coefficient of variation (CoV) calculated using the logarithmic method described by Bland and Altman [22]. In addition, inter-observer and intra-observer variability were also assessed using one-way intraclass correlation coefficient (ICC). Both CoV and ICC were displayed with their 95% confidence intervals. Qualitative and quantitative image scores were also compared using the paired t-test. This was done as previous work has shown that the paired t-test has a lower Type II error rate compared to non-parametric tests for Likert scale data [23]. It is therefore more likely to detect differences between the two techniques. A *p*-value of less than 0.05 indicated a significant difference.

## Results

### Demographics and feasibility

The mean age of population was 13.6 ± 2.7 years (median: 13.5, range: 7.0–18.3) and 33 (55%) were female. The mean heart rate was 82.3 ± 15.9 bpm (median: 82, range: 50–114). The full demographic information and patient diagnoses are shown in Table 2. Real-time and standard breath hold imaging was successfully performed in all subjects. The total time acquisition time for the full breath hold stack was 348 ± 79 s (median: 335, range: 239–573) compared to 19.8 ± 5.8 s for the acquisition of the real-time spiral stack (median: 19 s, range: 11–36). The online CS reconstruction for each real-time slice (all phases) was ~ 1.5 s, with an additional ~ 3.5 s per slice to send the data to the external computer and receive the reconstructed series back to the scanner database. In our buffered implementation, this resulted in all phases from all slices being available for viewing ~ 28 s after the end of the acquisition.

**Table 2:** Full demographic information and patient diagnoses

	Mean ± standard deviation (range)
Male/Female	27/33
Age (years)	13.6 ± 2.7 (7.0 to 18.3)
Height (m)	1.6 ± 0.2 (1.2 to 1.9)
Weight (kg)	47.5 ± 15.8 (23.1 to 82.0)
BSA	1.4 ± 0.3 (0.9 to 2.0)
Heart rate (bpm)	82 ± 16 (50 to 114)
Pulmonary Hypertension	11
Cardiomyopathy	10
Family history Sudden death/cardiomyopathy	9
Pulmonary artery stenosis	3
Coarctation of the aorta	4
Tetralogy of Fallot (repaired)	4
Transposition of the great arteries (repaired)	3
Atrial septal defect	3
Ventricular Septal Defect	2
Aortopathy	5
Left ventricular outflow tract obstruction	3
Atrio-ventricular valve dysfunction	3

BSA, body surface area

### Image quality

**Fig. 2:**
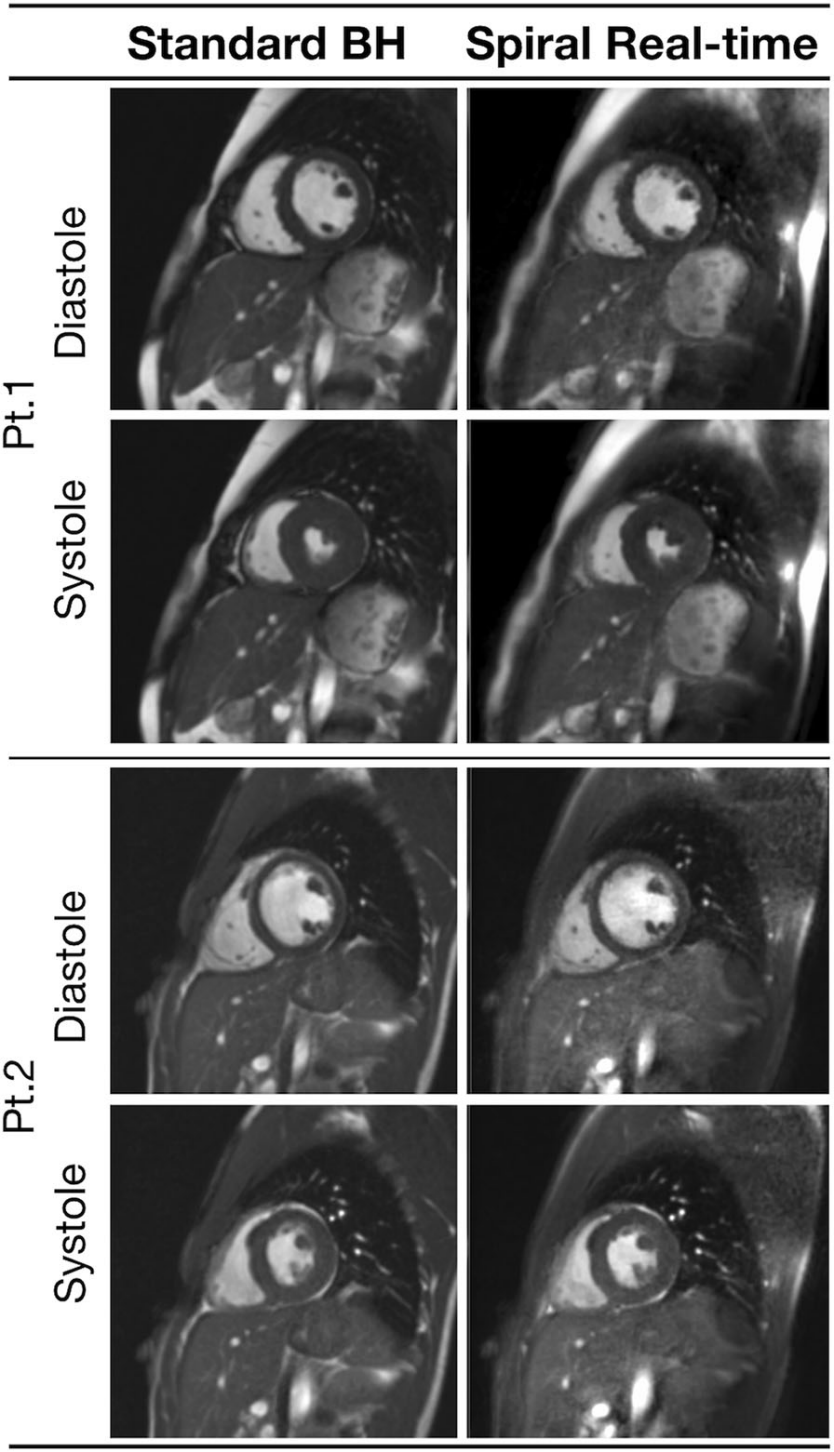
Example image quality from two patients with left heart disease. Example image quality at systole and diastole in two patients with congenital left heart disease. Pt1: Left ventricular outflow tract obstruction, Pt2: Family history of cardiomyopathy (see Additional file 1 and Additional file 2 for corresponding movies); BH=breath hold

**Fig. 3:**
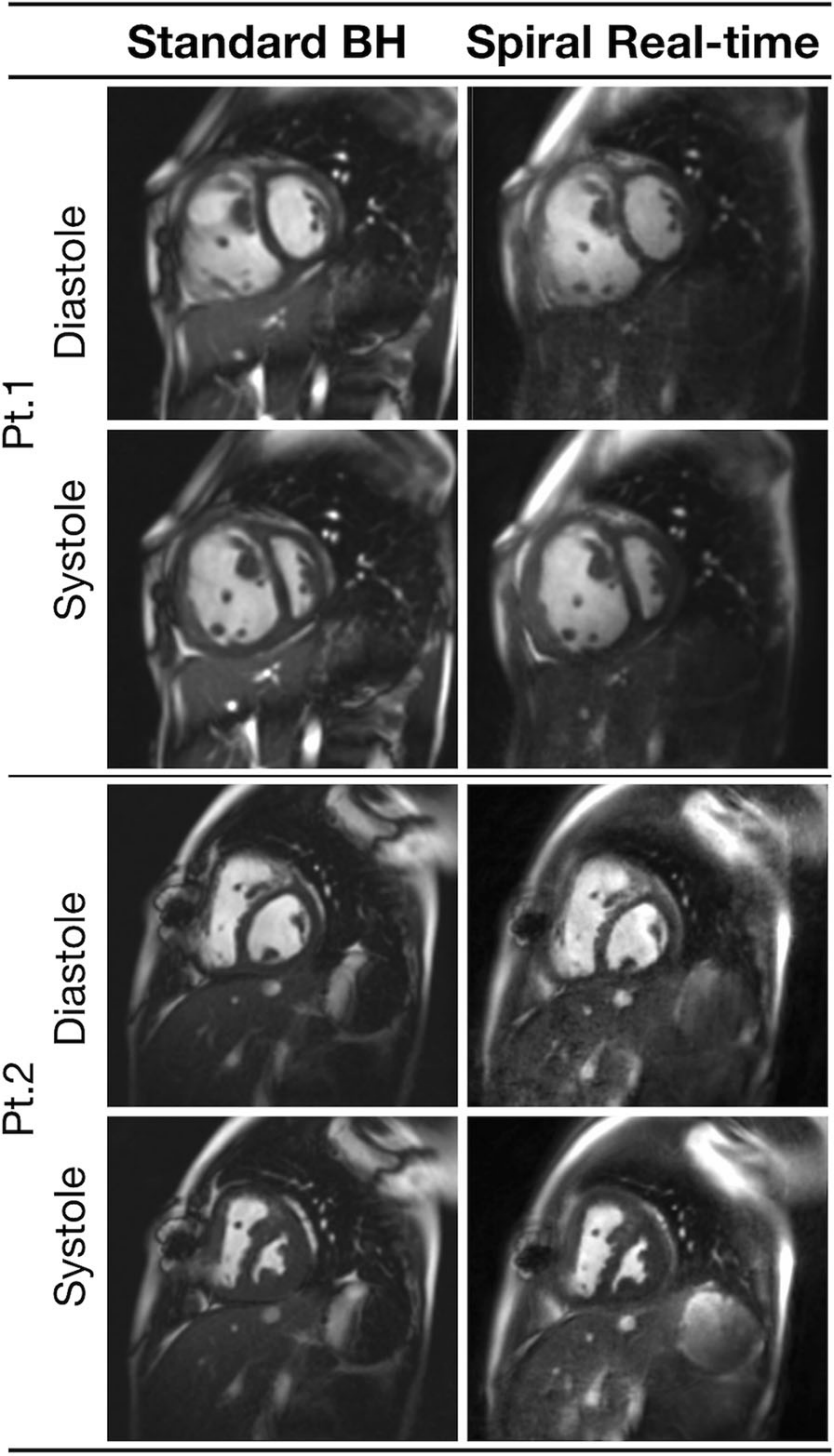
Example image quality from two patients with right heart disease. Example image quality at systole and diastole in two patients with congenital left heart disease. Pt1: Idiopathic Pulmonary Hypertension, Pt2: Double outlet right ventricle (see Additional file 3 and Additional file 4 for corresponding movies)

**Fig. 4:**
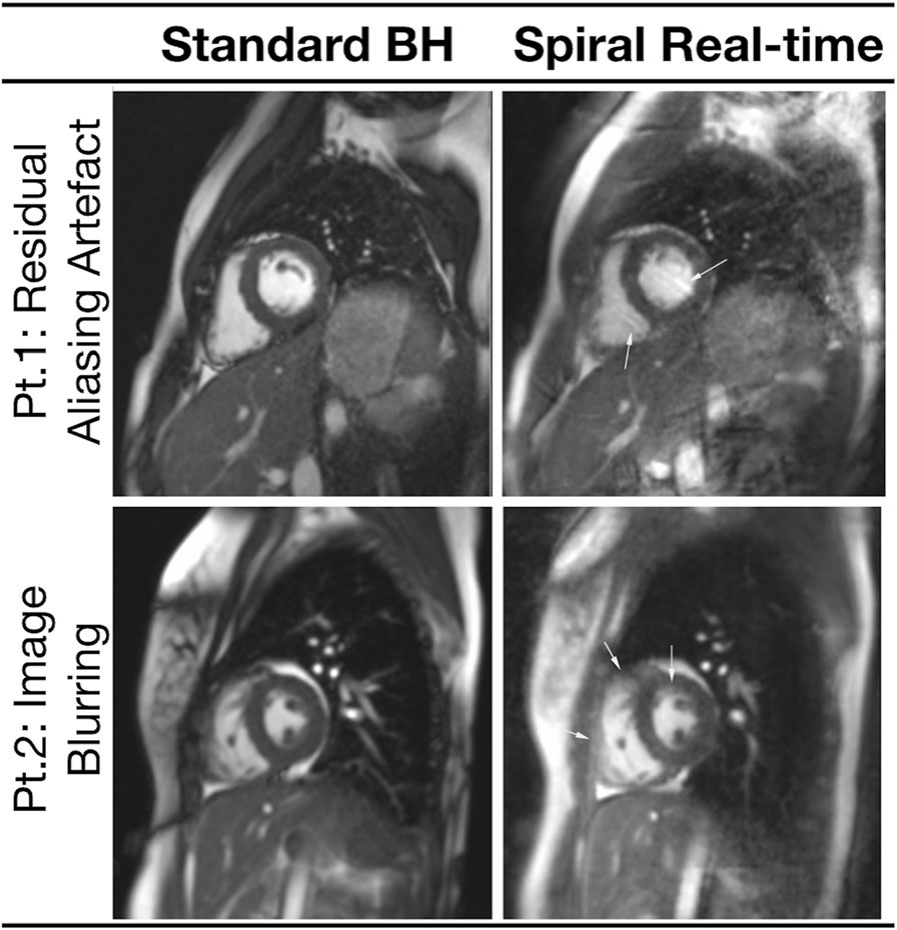
Example image quality from two patients with imaging artefacts. Example image quality in two patients with residual imaging artefacts. Arrows highlight artefacts on spiral real-time images. Pt1: Shows residual aliasing artefact, Pt2: Shows image blurring (see Additional file 5 and Additional file 6. for corresponding movies)

Representative images are shown in Figs. 2 and 3, and the corresponding movies can be seen in Additional files 1, 2, 3, and 4. These demonstrate that the spiral real-time images are of good quality. However, Fig. 4 shows examples of cases with residual aliasing artefacts and reduced edge definition (corresponding movies can be seen in Additional file 5 and Additional file 6), which is reflected in the qualitative image scores. Specifically, spiral real-time images had more residual aliasing artefacts than standard breath hold images (artefact score: 3.8 ± 0.1 vs 4.8 ± 0.1, *p* < 0.001) and lower edge definition (edge score: 4.4 ± 0.7 vs 4.8 ± 0.5, *p* < 0.001). There was a trend towards poorer motion fidelity (motion score: 4.6 ± 0.1 vs 4.8 ± 0.1, *p* = 0.06). There were no significant differences between qualitative image scores for the different observers (*p* > 0.1).

Quantitative edge sharpness was also significantly lower (*p* < 0.001) for the spiral real-time images compared to the standard BH images (0.68 ± 0.19 mm^− 1^ vs 0.53 ± 0.16 mm^− 1^, respectively). Additionally, quantitative estimates of image contrast (blood pool-to-myocardial signal intensity ratio) were significantly lower (*p* < 0.001) for the spiral real-time images compared to the standard BH images (2.7 ± 0.4 vs. 3.2 ± 0.4, respectively).

### Ventricular volume quantification

**Fig. 5:**
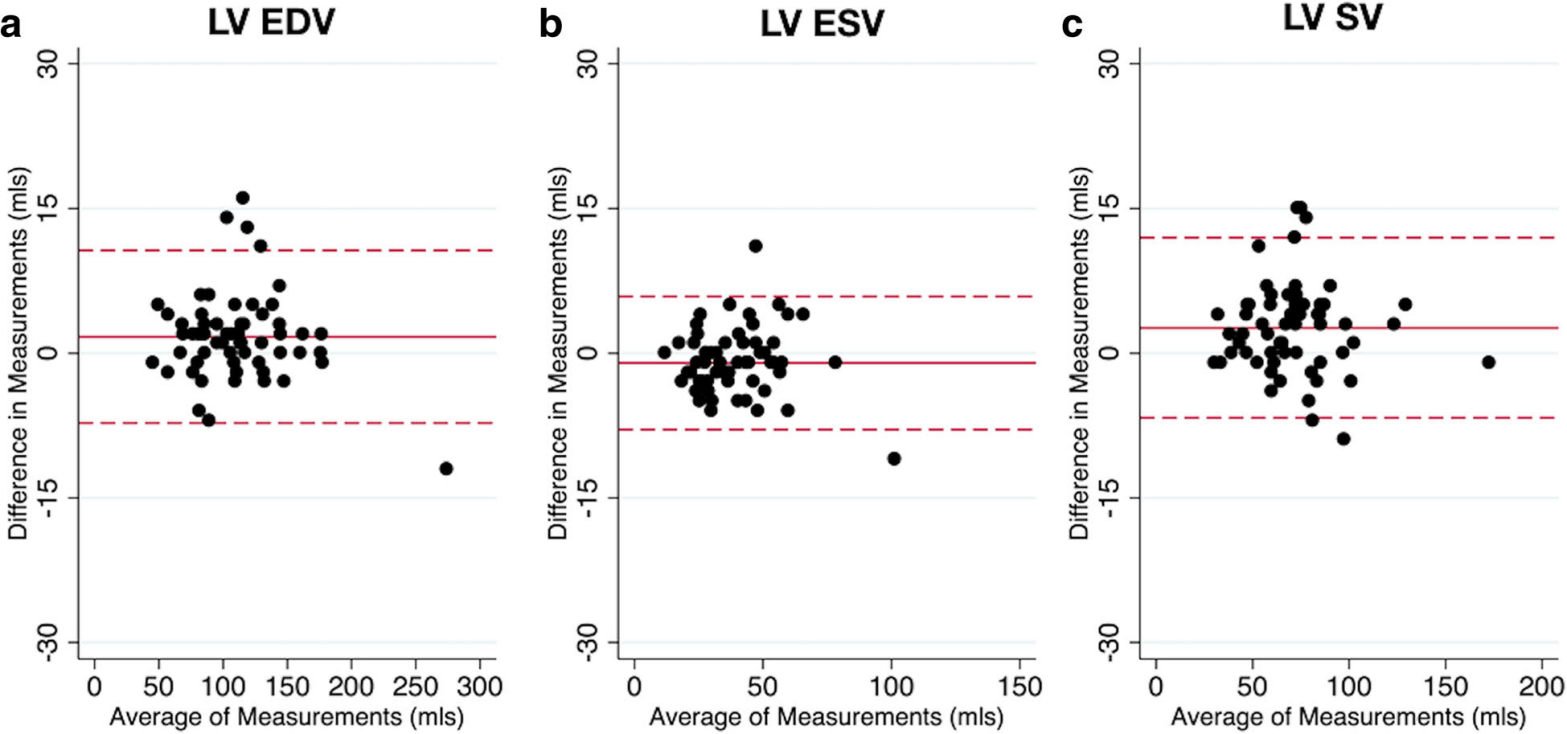
Left ventricular (LV) Bland-Altman plots. Bland-Altman plots of standard breath-hold (BH) vs real-time techniques for left ventricular (**a**) end-diastolic volume (EDV), (**b**) end-systolic volume (ESV), (**c**) stroke volume (SV). The solid red line indicates the bias, with the dashed red lines showing the upper and lower limits of agreement (bias±1.96xStandardDeviation) between the two techniques

**Fig. 6:**
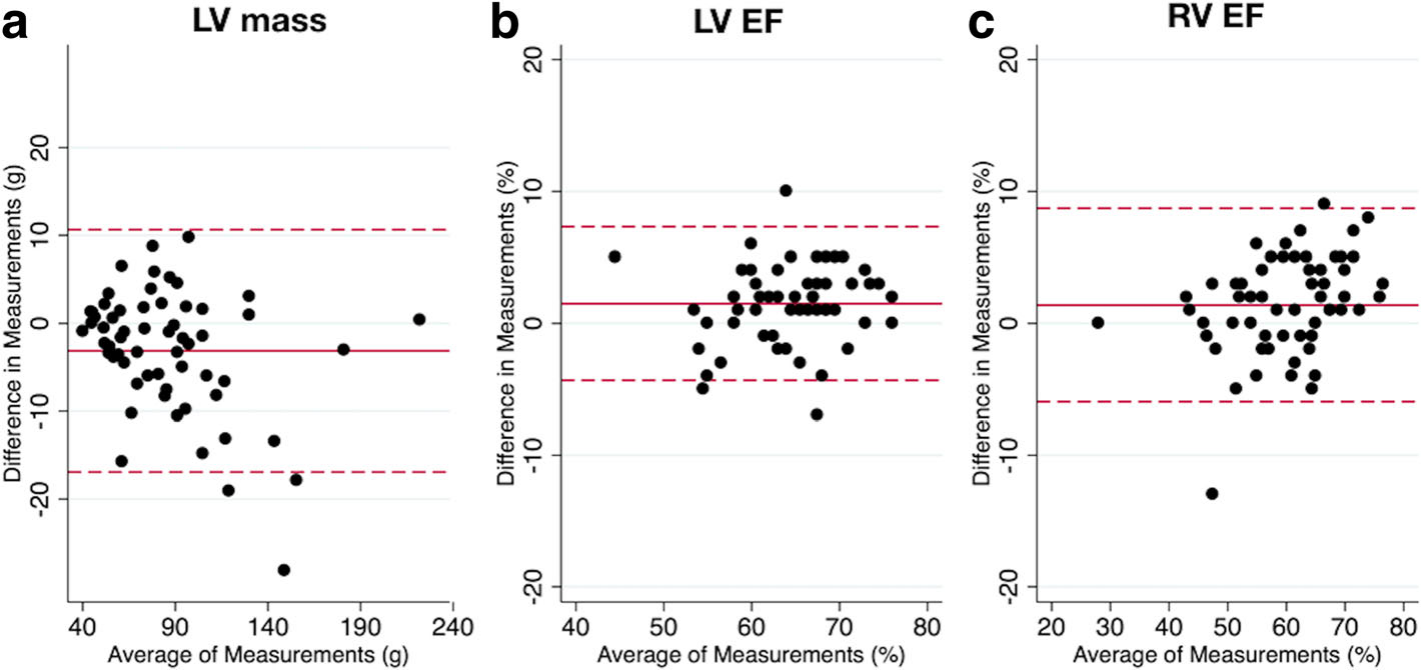
Bland-Altman plots for LV ejection fraction (EF) and LV mass. Bland-Altman plots of standard BH vs real-time techniques for; (**a**) left ventricular mass, (**b**) left ventricular ejection fraction (EF), (**c**) right ventricular EF. The solid red line indicates the bias, with the dashed red lines showing the upper and lower limits of agreement (bias±1.96xStandardDeviation) between the two techniques

Ventricular metrics measured using spiral real-time and clinical standard breath hold imaging are shown in Table 3. Bland-Altman plots for LV metrics are shown in Figs. 5 and 6. There was a small (1.7 mL) but significant underestimation of LV EDV, and a small (1.0 mL) but significant overestimation of LV ESV using the real-time imaging technique. This resulted in small underestimations of LV SV (2.6 mL) and LVEF (1.5%). There was also a small but significant overestimation of LV mass (3.1 g). Nevertheless, the limits of agreement between real-time and breath hold data for all LV metrics were narrow. Body surface area correlated with the percentage difference between real-time and breath hold derived LV mass (rho = − 0.27, *p* = 0.041). Differences for all other LV metrics were not associated (*p* > 0.05) with either heart rate or body surface area.

**Table 3:** Primary observer; ventricular metrics measured using real-time and standard BH imaging

	Real-time^a^	Standard Breath Hold^a^	Bias	Limits of agreement
LV EDV (mL)	110 ± 38	112 ± 38^b^	1.7 ± 4.6	−7.3 to 10.7
LV ESV (mL)	40 ± 16	39 ± 16^b^	− 1.0 ± 3.5	−7.9 to 5.9
LV SV (mL)	70 ± 25	73 ± 24^b^	2.6 ± 4.8	−6.7 to 12.0
LV EF (%)	64 ± 6	66 ± 7^b^	1.5 ± 3.0	−4.3 to 7.3
LV mass	88 ± 36	85 ± 34^b^	− 3.1 ± 7.0	− 16.9 to 10.6
RV EDV (mL)	121 ± 37	121 ± 38	0.5 ± 5.2	−9.6 to 10.6
RV ESV (mL)	51 ± 23	49 ± 24	− 1.2 ± 5.0	−11.0 to 8.5
RV SV (mL)	70 ± 20	72 ± 20^b^	1.8 ± 4.9	−7.8 to 11.4
RV EF (%)	59 ± 9	61 ± 10^b^	1.4 ± 3.7	−6.0 to 8.7

*EDV, end-diastolic volume; EF, ejection fraction; ESV, end-systolic volume, LV, left ventricular; RV, right ventricular; SV, stroke volume*

^a^*Displayed as mean* ± standard deviation

^b^ *Indicates significant differences with Standard BH technique (p < 0.05)*

*Bias is the mean of the paired difference (Standard BH – Real-time) presented with standard deviation (SD) of the difference*

*Limits of agreements are bias* ± 1.96xSD

**Fig. 7:**
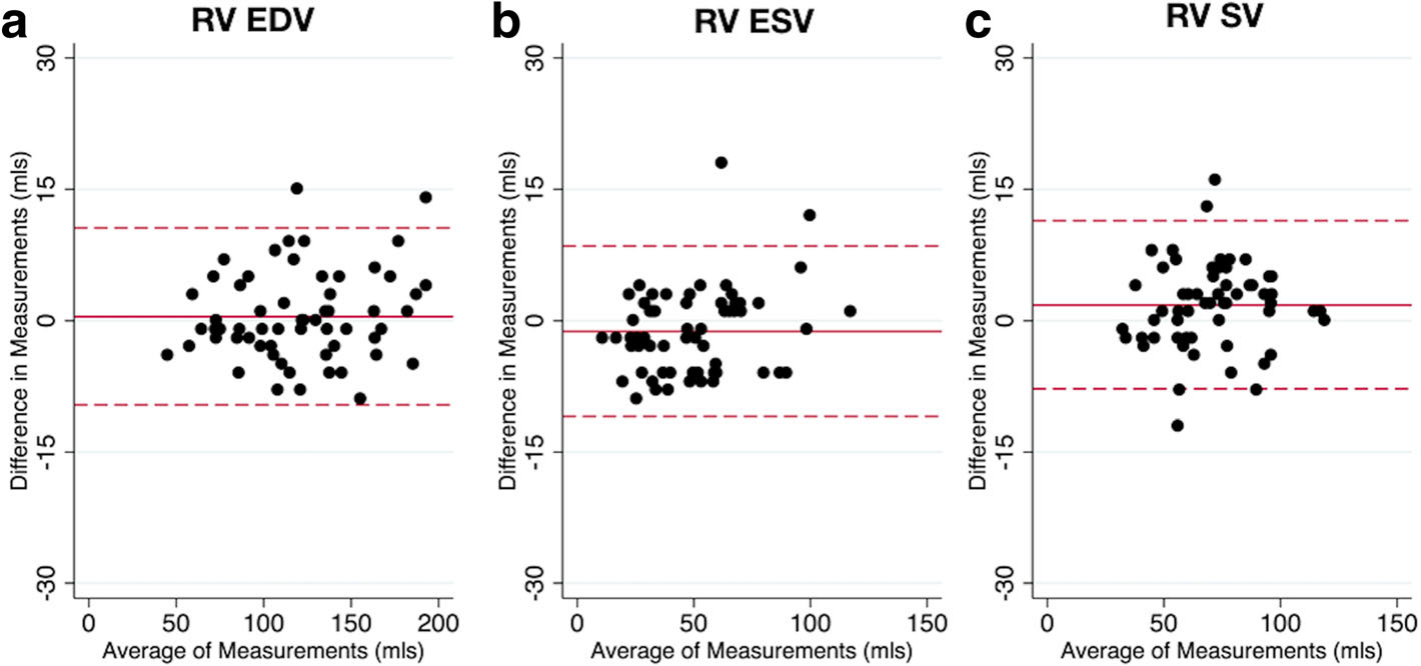
Right ventricular Bland-Altman plots. Bland-Altman plots of standard breath hold vs real-time techniques for right ventricular; (**a**) EDV, (**b**) ESV, (**c**) SV. The solid red line indicates the bias, with the dashed red lines showing the upper and lower limits of agreement (bias±1.96xStandardDeviation) between the two techniques

Bland-Altman plots for RV metrics are shown in Figs. 6 and 7. There were no significant differences in RV EDV or ESV, however there was a small underestimation of RV SV (1.8 mL) and RV EF (1.4%). However, the limits of agreement between real-time and BH data for all RV metrics were narrow. Differences in RV metrics were not significantly (*p* > 0.05) associated with either heart rate or body surface area.

The inter-observer CoV’s and ICC’s are shown in Table 4 and intra-observer CoV’s and ICC’s are shown in Table 5. The largely overlapping confidence intervals demonstrated that there were no significant differences in inter-observer and intra-observer variability between spiral real-time and standard breath hold derived RV and LV metrics.

**Table 4:** Inter-observer variability; ventricular metrics measured using real-time and standard BH imaging

	Standard Breath Hold CoV^a^	Real-time CoV^a^	Standard Breath Hold ICC^a^	Real-time ICC^a^
LV EDV (%)	4.1 (3.1 to 5.2)	4.0 (2.9 to 5.0)	0.983 (0.964 to 0.992)	0.984 (0.968 to 0.992)
LV ESV (%)	7.0 (5.2 to 8.9)	8.7 (6.4 to 11.0)	0.973 (0.946 to 0.987)	0.931 (0.862 to 0.967)
LV SV (%)	7.1 (5.3 to 9.0)	6.8 (5.1 to 8.6)	0.958 (0.914 to 0.980)	0.952 (0.902 to 0.977)
LV EF (%)	4.2 (3.1 to 5.3)	5.7 (4.2 to 7.2)	0.891 (0.785 to 0.946)	0.772 (0.578 to 0.884)
RV EDV (%)	6.6 (4.9 to 8.3)	6.9 (5.1 to 8.8)	0.964 (0.926 to 0.983)	0.956 (0.911 to 0.979)
RV ESV (%)	13.2 (9.7 to 16.8)	12.8 (9.4 to 16.3)	0.968 (0.935 to 0.985)	0.954 (0.906 to 0.978)
RV SV (%)	10.9 (8.0 to 13.9)	11.5 (8.5 to 14.6)	0.865 (0.738 to 0.933)	0.835 (0.684 to 0.917)
RV EF (%)	7.1 (5.3 to 9.0)	7.7 (5.7 to 9.7)	0.836 (0.687 to 0.918)	0.785 (0.599 to 0.891)

^a^ *Displayed as mean (95% confidence intervals)*

**Table 5:** Intra-observer variability; ventricular metrics measured using real-time and standard breath hold imaging

	Standard Breath Hold CoV^a^	Real-time CoV^a^	Standard Breath Hold ICC^a^	Real-time ICC^a^
LV EDV (%)	1.7 (1.3 to 2.1)	1.5 (1.1 to 1.9)	0.994 (0.987 to 0.997)	0.997 (0.994 to 0.999)
LV ESV (%)	4.3 (3.2 to 5.5)	4.7 (3.5 to 6.0)	0.985 (0.968 to 0.993)	0.984 (0.967 to 0.992)
LV SV (%)	2.3 (1.7 to 2.9)	2.6 (1.9 to 3.2)	0.993 (0.985 to 0.997)	0.994 (0.988 to 0.997)
LV EF (%)	1.8 (1.4 to 2.3)	2.4 (1.8 to 3.0)	0.955 (0.909 to 0.978)	0.919 (0.839 to 0.961)
RV EDV (%)	2.0 (1.5 to 2.5)	2.2 (1.6 to 2.7)	0.994 (0.987 to 0.997)	0.994 (0.987 to 0.997)
RV ESV (%)	4.2 (3.1 to 5.3)	5.0 (3.7 to 6.3)	0.989 (0.977 to 0.995)	0.977 (0.952 to 0.989)
RV SV (%)	3.6 (2.6 to 4.5)	4.2 (3.1 to 5.3)	0.989 (0.977 to 0.995)	0.985 (0.968 to 0.993)
RV EF (%)	2.7 (2.0 to 3.4)	3.4 (2.5 to 4.3)	0.975 (0.949 to 0.988)	0.946 (0.890 to 0.974)

^a^ *Displayed as mean (95% confidence intervals)*

## Discussion

The main findings of this study were that: i) real-time bSSFP imaging using tiny golden-angle spiral trajectories combined with a CS reconstruction was feasible, ii) the image quality of the real-time technique was slightly lower than the standard breath hold technique, iii) there was good agreement for quantification of both LV and RV metrics between the spiral real-time and breath hold clinical standard techniques.

### Spiral bSSFP sequence with CS reconstruction

Real-time CMR is particularly useful when imaging children, as it can be performed quickly and without breath holds. In this study, we implemented a novel real-time sequence that leveraged both efficient spiral *k*-space filling and CS reconstruction. Spiral *k*-space filling has previously been used to successfully accelerate spoiled gradient echo sequences (i.e. phase contrast CMR [24] and CMR angiography [25]). However, there are some problems associated with combining spiral trajectories with bSSFP readouts [14, 26, 27]. These mainly relate to off-resonance effects and longer repetitions times. Previous studies have shown that these effects can be partly mitigated using both zeroth and first order moment rewinders to balance the imaging gradients [14, 28]. The problem with this approach is that it significantly increases repetition time and lowers temporal resolution. Therefore, we chose to use a shorter conventional zeroth order moment rewinder, combined with a relatively short readout (1.86 ms) to reduce off-resonance effects. However, this increases the number of interleaves required to fully sample *k*-space. Consequently, high acceleration factors were required to ensure adequate temporal resolution and we utilized CS to reconstruct artefact-free images. Our CS implementation shares many characteristics with the previously validated GRASP reconstruction for radial trajectories [18]. Specifically, we utilized tiny golden angle spacing to produce temporal incoherency and penalized temporal finite differences in the reconstruction. Combining spiral bSSFP with CS resulted in a real-time sequence with only slightly lower spatial resolution, and similar temporal resolution, to clinical standard breath hold imaging.

### Clinical utility

Clinical utility depends on the ability of the reader to process the images to accurately measure ventricular volumes. In this study, images were processed by three independent CMR specialists (not involved in the development of the sequence) This is a more ‘real-world’ test of the sequence, in contrast to many studies in which a single observer (often involved in the development of the technology) is used.

We demonstrated that spiral real-time images were of diagnostic quality. However, they did have slightly lower edge definition and greater amount of residual aliasing artefact. This is not surprising as the real-time images had lower spatial resolution and were acquired with significant acceleration. Interestingly, although CS with temporal finite difference sparsity can result in temporal blurring, there was only a trend towards reduced motion fidelity.

Irrespective of differences in image quality, from a clinical point it is the ability to accurately measure ventricular volumes that is paramount. In this study, we found that LV EDV was smaller and LV ESV was bigger with real-time imaging compared to standard BH imaging. We also found similar differences in RV ESV, although this did not reach statistical significance. These biases were probably due to inaccurate segmentation of the real-time images, secondary to lower edge sharpness and myocardial blood pool contrast. In diastole, we believe these inaccuracies resulted in blood pool being included in the myocardial mass and EDV being underestimated. We believe the converse occurred in systole, with myocardial mass being included in the blood pool and ESV being overestimated. An important question in children, is whether the differences between real-time and standard breath hold imaging were associated with heart rate or body size. In this study, the only significant association we found was between percentage overestimation of LV mass and decreasing BSA. This was probably due to errors in epicardial segmentation in smaller children, possibly due to factors such as less pericardial fat and geometric considerations. Nevertheless, the biases were all below 5% and would be expected to have minimal effect on clinical decision making. More importantly, the limits of agreement were narrow compared to previously described real-time methods (including those that utilize CS) [6, 29]. We believe that this is due to the high spatial and temporal resolution of our spiral real-time sequence, which was only slightly lower than the standard breath hold sequence. In this study, we also examined inter-observer and intra-observer variability (half the populations tested for each). For the standard breath hold sequence the coefficients of variation and ICC’s were similar to previously published data [30], with RV data having greater variability than LV data. This is to be expected due to the greater difficulty in segmenting the more trabeculated and complex shaped RV. Importantly, the inter-observer and intra-observer variability of real-time sequences were similar to standard breath hold imaging. This is an important finding, as demonstrating reliability is vital for clinical translation. We believe that our findings show that spiral real-time imaging can successfully be used for assessment of paediatric heart disease. The main benefit over standard breath hold imaging is that it can be acquired during free breathing. Another important secondary benefit is that a whole ventricular stack can be acquired in approximately 20 s, as opposed to almost 6 min for standard breath hold imaging. Thus, this technique holds the potential to significantly shorten CMR scan times in children.

### Limitations

The main limitation of this study was the minimum age in the population was 7 years, which limits the translatability of our findings to younger children and infants. The reason for this age limit was that younger children and infants would have found it difficult to perform the standard breath hold sequence necessary for validation. Nevertheless, future studies concentrating on optimization and validation of this technique for infants would be useful. Another limitation was that we did not assess RV mass in this study. Several studies have suggested that RV mass may be an important predictive marker in certain diseases [31]. However, RV mass is difficult to accurately quantify in patients without RV hypertrophy, even when using standard breath hold imaging. Therefore, we did not include it this study, although it could be included in future studies concentrating on patient with RV disease. It should also be noted that the real-time imaging was performed during free breathing and physiological variation may account for some discrepancies with standard breath hold imaging. However, the differences probably remain small because the slices in the real-time short axis stack are all acquired at in different points in the respiratory cycle. Thus, any discrepancies may ‘average out’ when summed across the whole ventricle. In terms of intra-observer variability, there was no prescribed time delay between processing of data, as volumes (including repeated volumes) were presented data in a randomized manner. However, both the standard breath hold and real-time data was treated in the same manner and the variability measures were in keeping with previously published data. Thus, we believe the similar intra-observer variability of the real-time and standard breath hold data does reflect the clinical reality. Finally, one disadvantage of compressed sensing is long reconstruction times. In this study, we used an online GPU based reconstruction that fed images back to the scanner in a clinically acceptable time frame (< 30 s for the entire short axis stack). Nevertheless, more work is required so that reconstruction times can be reduced to that of standard Cartesian imaging.

## Conclusion

In conclusion, we implemented a novel spiral real-time bSSFP sequence with CS reconstruction. We showed good agreement between biventricular volumes measured using spiral real-time and standard breath hold techniques in children with heart disease. Thus, we believe that this technique could be used successfully in the paediatric population.

## Additional files

Additional file 1: **Movie 1:** Cine showing image quality in one patient with left heart disease: Left ventricular outflow tract obstruction. (MOV 2013 kb)

Additional file 2: **Movie 2:** Cine showing image quality in one patient with left heart disease: Family history of Cardiomyopathy. (MOV 3451 kb)

Additional file 3: **Movie 3:** Cine showing image quality in one patient with right heart disease: Idiopathic Pulmonary Hypertension. (MOV 3015 kb)

Additional file 4: **Movie 4:** Cine showing image quality in one patient with right heart disease: Double outlet right ventricle. (MOV 1885 kb)

Additional file 5: **Movie 5:** Cine showing residual aliasing artfeact in the spiral real-time images in one patient. (MOV 2454 kb)

Additional file 6: **Movie 6:** Cine showing residual blurring in the spiral real-time images in one patient. (MOV 1990 kb)

## Abbreviations

BH: Breath-hold
BSA: Body surface area
bSSFP: Balanced steady state free precession
CMR: Cardiovascular magnetic resonance
CNR: Contrast-to-noise ratio
CoV: Coefficient of variation
CS: Compressed sensing
EDV: End-diastolic volume
EF: Ejection fraction
ESV: End-systolic volume
GRASP: Golden-angle Radial Sparse Parallel
ICC: Intra-class correlation
LV: Left ventricle/left ventricular
RV: Right ventricle/right ventricular
SD: Standard deviation
SNR: Signal-to noise ratio
SV: Stroke volume
tGA: Tiny Golden Angle
TR: Repetition time
